# Portable Bioactive Paper-Based Sensor for Quantification of Pesticides

**DOI:** 10.1155/2013/932946

**Published:** 2013-07-22

**Authors:** Murat Kavruk, Veli Cengiz Özalp, Hüseyin Avni Öktem

**Affiliations:** ^1^NanoBiz Ltd. Metu Technopolis, Galyum Block, Floor 2, No. 18, 06800 Ankara, Turkey; ^2^Department of Biology, Nanobiotechnology R&D Lab, Middle East Technical University, 06800 Ankara, Turkey; ^3^School of Medicine, Istanbul Kemerburgaz University, 34217 Istanbul, Turkey

## Abstract

A paper-based biosensor was developed for the detection of the degradation products of organophosphorus pesticides. The biosensor quantifies acetylcholine esterase inhibitors in a fast, disposable, cheap, and accurate format. We specifically focused on the use of sugar or protein stabilizer to achieve a biosensor with long shelf-life. The new biosensor detected malathion with a detection limit of 2.5 ppm in 5 min incubation time. The operational stability was confirmed by testing 60 days storage at 4°C when glucose was used as stabilizer.

## 1. Introduction

Detection of pesticide traces in food and water is an important safety issue due to intensive agricultural applications and their consequent toxicity. Pesticides, such as organophosphates (OP) and carbamates (CM), have inhibitory effects on cholinesterases which are enzymes essential for the proper functioning of the nervous system of vertebrates and insects. The toxic action of organophosphate and carbamates arise from the inhibition of acetylcholinesterase activity leading to accumulation of acetylcholine at the nerve endings and therefore causing cholinergic overstimulation characterized by severe consequences in humans including abdominal cramps, muscular tremor, hypotension, breathing difficulty, diarrhea, slowing heartbeat (bradycardia), muscular fasciculation, and paralysis [1]. Therefore, portable and accurate quantification of pesticides is essential for public health.

The detection of pesticides or nerve agents has been traditionally carried out in laboratory settings with large and expensive instruments such as gas chromatography coupled with mass spectroscopy (GC-MS) [2], spray mass spectroscopy [3], or high performance liquid chromatography (HPLC) [4]. Recent research efforts focused on developing biosensors platforms that can be incorporated into mobile detection devices. In that respect, paper attracts considerable attention as a matrix for developing low-cost analytical devices [5]. Paper is affordable, abundant, disposable, and has high volume to surface ratio. Paper-based biosensors are usually fast-responding and low-cost diagnostic tools in health and environmental applications. Bioactive papers are obtained by modification of paper matrix with biomolecules in order to add sensor functionality. One of the major advantages of bioactive paper sensors is that they are designed to operate without sophisticated equipment [6]. Generally the sensitivity is tolerated at the expense of simple applications compared to other analytical methods. In bioactive paper biosensors, enzyme-immobilized paper is the matrix for fluid sample transportation, biological detection, and the detection in a single step process. For paper-based biosensors, a variety of colorimetric formats have been developed including dipstick techniques and lab-on-paper microfluidic systems.

Acetylcholine is a neurotransmitter active in central nervous systems and skeletal-muscle junction. Acetylcholine esterase (AChE) is the hydrolase that degrades acetylcholine molecules into choline and acetic acid, thus terminating impulse transmission at cholinergic synapsis. Therefore, AChE controls generation of nerve impulses in the postsynaptic neurons. Toxicity of OP and CM depends on inhibition of AChE; thus the enzyme is a common bioevaluator for the detection of organophosphates and carbamates [7]. Extention of inhibition of this enzyme has been frequently used to measure quantitatively the presence of such pesticides. AChE inhibition has been proven to be useful in monitoring organophosphate and carbamate changes in samples with various platforms of sensors including surface plasmon resonance (SPR) [8], electrochemical [9, 10], colorimetric [11], various nanomaterials-based methods [12–15]. However, AChE is the most widely used biorecognition element in biosensor development for pesticide detection [7]. Paper matrix has been the improved material for some AChE-based detection devices such as sol-gel entrapment of gold nanoparticles for paper-dipstick sensor device [16], a lateral flow application [17]. Moreover, microfluidic paper devices could be developed by patterning hydrophilic channels and hydrophobic barriers [18]. Thus, paper could be a low-cost ubiquitous material for developing alternative multiplex sensors for onsite pesticide determination for medical diagnostics, environmental monitoring, or food quality analysis.

In this study, a paper-based sensor was developed as a rapid and reliable monitoring method for organophosphates and carbamates. The model interaction between malathion and its specific inhibition of AChE was used to monitor quantitative changes in order to develop a mobile biosensor. Unlike the previously reported acetylcholinesterase-based paper biosensors, we evaluated sugar and protein stabilizers in order to develop a biosensor with improved shelf-life.

## 2. Materials and Methods

### 2.1. Materials

AChE (from electric eel), (5,5′-dithiobis-(2-nitrobenzoic acid) (DTNB), acetylthiocholine iodide (ATCh), and malathion were purchased from Sigma-Aldrich. The chemicals used in the preparation of buffers were purchased from Merck. Munktell No. 1 filter discs were purchased from Munktell (Falun, Sweden).

### 2.2. Biosensor Construction

**Figure 1:**
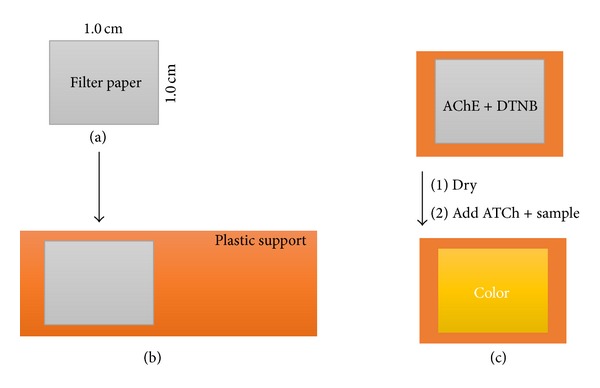
Schematic representation of biosensor support construction. (a) Munktell filter papers were cut and (b) fixed on a plastic support. (c) The enzyme mixture (AChE and DTNB) was directly applied on the fixed paper and dried. The samples with ATCh were directly applied on dried paper strips for color formation.

A mixture of enzyme, substrate, chromophore, and stabilizer solution was prepared for the indicated final concentrations in each experiment. For preparing biosensor strips, Munktell filter paper discs were prepared in 1 × 1 cm pieces and autoclaved at 120°C for 25 minutes prior to use (Figure 1(a)). Filter papers were then fixed via two-sided plaster on plastic supports (Figure 1(b)). A mixture of AChE and DTNB solution of 15 *μ*L was then applied on the filter paper by direct pipetting and drying in a desiccator at −600 mmHg for 20 minutes. Subsequently, 15 *μ*L of ATCh solution was directly applied on the filter paper that contains enzyme-DNTB mixture. The samples were included in the ATCh solutions without changing the final volume. The color intensity was measured at exactly 5 minutes after the addition of ATCh containing solution (Figure 1(c)).

### 2.3. Cholinesterase Inhibition Assay

The ChE inhibition assay is based on quantification of free sulfhydryl groups of thiocholine which is the product of acetylthiocholine hydrolyzation by acetylcholine esterase. Ellman's reagent, 5,5-dithiobis-(2-nitrobenzoic acid) (DTNB), is used to generate a yellow chromophore detectable at the 405 nm [19]. The color development is at maximum level at the beginning, and the enzyme inhibitor addition reduces the final color developed. Color development change was converted to inhibitor concentration by visual comparison or spectrophotometric measurements using malathion as standard inhibitor. pH studies were carried out between 1.0 and 13.0 with 1.0 grade increments. Effect of temperature was investigated at 4°C, 25°C, and 37°C to determine optimum condition for the biosensor to work efficiently. In the shelf-life experiment, 4°C and RT conditions were tested. In all temperatures, biosensors were incubated at that specific temperature for 5 minutes after dropping of ATChI solution. Malathion was used as an inhibitor of enzyme and target molecule of the biosensor. Up to 200 ppm malathion was investigated to determine the detection limit of the biosensor. The stability of the biosensor was compared in the presence of glucose (up to 15% w/v), trehalose (up to 8% w/v), or BSA (up to 5% w/v) in order to improve the stability of the enzyme AChE. The stabilizers were added in the AChE-DNTB mixture and final volume was always 15 *μ*L. The digital pictures of the sensor measurements were obtained by putting the filter papers between two acetate papers and scanning them via Canon Pixma MP610 multifunctional scanner with time intervals. These digital pictures were converted into numerical values.

## 3. Results and Discussions

**Figure 2:**
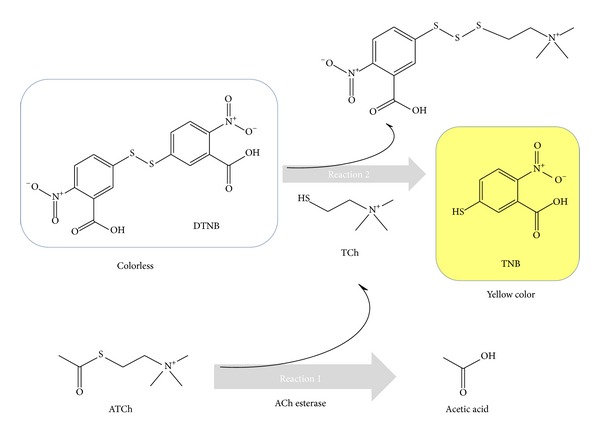
Two-step sequential reactions of acetylthiocholine (ATCh) for production of yellow colored TNB. In reaction 1, ATCh is broken to acetic acid and thiocholine (TCH). The free sulfhydryl group of TCh is quantified through Ellman's method in reaction 2. The resulting TNB is used in a direct determination of the activity of ACh esterase.

In this study, a paper-based sensor device was developed as a rapid and reliable monitoring method for OP and CM pesticides. The device is composed of dipstick paper sensor and a CCD camera for analysis in a portable format. The model interaction between malathion and its specific inhibition of AChE was used to monitor quantitative performance of the sensor. A biosensor for AChE inhibitory molecules has been developed by immobilizing the enzyme, its substrate (ATCh), and a chromophore (DTNB; 2-nitro-5-thiobenzoic acid) in paper matrix through adsorption. The chemical reactions leading to inhibitor dependent colour development are summarized in Figure 2. Acetylcholine esterase inhibition can be quantified by an assay method first described by Ellman et al. [19]. DTNB is a water soluble chemical that can specifically react with free sulfhydryl groups. AChE hydrolyses ATCh to produce thiocholine (TCh) and acetate. Then, the free sulfhydryl group of TCh reduces DTNB to TNB which is absorbed at 405 nm. The difference between initial AChE activity and that after incubation with inhibitors is used to calculate the concentration of inhibitory molecules.

**Figure 3:**
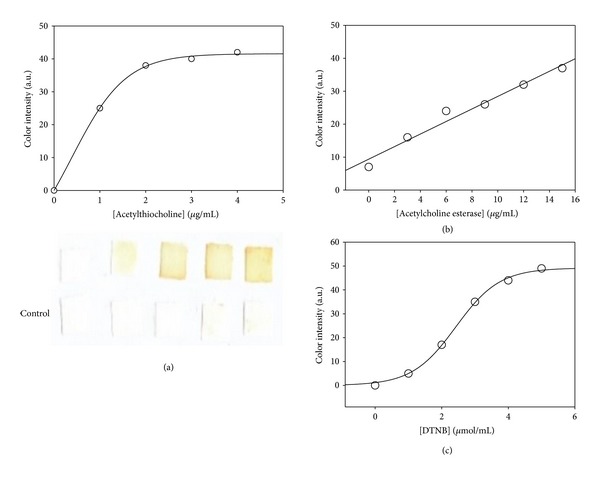
The response of biosensor (a) at different substrate, (b) enzyme, or (c) Ellman's reagent concentrations. In each experiment, ATCh, AChE, and DTNB were mixed and adsorbed into paper matrix. Their fixed concentrations were AChE 12 U/mL, DTNB 4 *μ*g/mL, and ATChI 4 *μ*g/mL. The lower panel in (a) shows pictures of paper sensor; upper row is performed with sensor mixture and lower row with control mixture (lacking the enzyme component).

To determine the working amounts of sensing components, a range of concentrations for each of them was systematically optimized for the best combination of AChE as the enzyme, DTNB as the chromophore, and ATChI as the artificial substrate. Optimum concentrations were selected according to the quantified colour production and the observation based on the naked eye, since occasionally the proposed biosensor would be used in the field with minimum instrumental help.  Figure 3 shows that increasing amount of biosensor components generally resulted in higher colour development as expected. For the determination of the optimal DTNB concentration, up to 5 *μ*g/mL final concentrations were tested for color development with fixed amount of 12 U/mL enzyme and 4 *μ*g/mL ATChI. A higher density in yellow colour formation was observed with an increasing ATChI concentration (Figure 3(a)). Experiments with 4 *μ*g/mL gave a distinguishable yellow colour from control samples and had a response time faster than the other concentrations. Thus, 4 *μ*g/mL of DTNB as the final concentration for the chromophore in the final biosensor was chosen based on this data.  Figure 3(b) is an enzyme titration curve when DTNB and ATChI were fixed at 4 *μ*g/mL. The colour intensity was directly proportional to the amount of enzyme for all concentrations used in this study. Subsequently, we tested ATChI for best signal by fixing DTNB concentration at 4 *μ*g/mL and 12 U/mL enzyme.  Figure 3(c) shows that 4 *μ*g/mL DTNB resulted in a biosensor with more rapid color formation. The overall results from these experiments resulted in the identification of optimum concentrations of biosensor mixture at, (i) 4 *μ*g/mL ATChI, (ii) 4 *μ*g/mL DNTB and (iii) 12 U/mL AChE. All subsequent experiments were conducted using there optimized concentrations of biosensor components.

**Figure 4:**
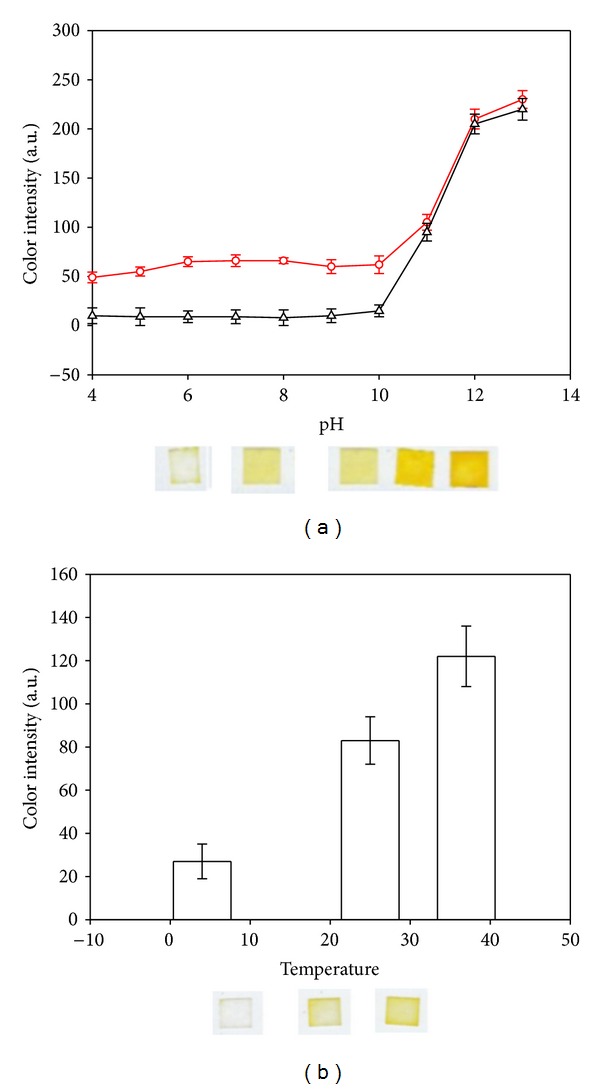
(a) The response of biosensor at different pH values from 4 to 13. Red line (O) is the color intensity of DNTB and ATCh with AChE and black line (Δ) represents the color intensity without AChE. (b) Effect of temperature on biosensor response. Vertical bars indicate standard error of mean.

The effect of pH on the biosensor platform was investigated between 1 and 13 (Figure 4) since real samples could have components changing acidity. There was a slight response between the values of pH 2 to pH 8. Due to a small activity loss or the degradation of the enzyme, at pH 9, slightly less yellow colour formation was obtained. At alkaline values more than 10, the yellow colour formation was instant after pouring the ATChI on to the enzyme-chromophore stabilized part, and the density of the yellow increased immensely. This can be explained by cleavage of either DTNB or the ATChI. Therefore, the sensor would require well-buffered treatments especially for strongly alkaline samples.

Thermal stability of AChE on paper was measured by an investigation at temperatures 4°C, 25°C, and 37°C. For all temperatures, the biosensors were incubated at that specific temperature for 5 minutes after the addition of ATChI solution to the mixture of other components. Temperature studies showed that at 4°C the activity of the enzyme ceased and no colour formation was observed. At room temperature and at 37°C, the yellow colour formation occurred with significantly different colour units with respect to control sensors. 

Enzyme-based biosensors have been known for their selectivity, specificity, and catalytic signal amplification for the development of biosensors [20, 21]. However, the applications of enzyme-based devices remain limited mostly due to the liability for extreme physical conditions (e.g., pH, high temperature, and shear force). Thus, special procedures are required for increasing their stability during storage. Immobilized enzymes can exhibit much better functional properties when immobilization strategies are properly designed for the enzyme [22]. In our biosensor, the stability of the AChE is the most labile part of the biosensor; the other important drawback is mentioned to be the instability of the artificial substrate ATCh. The activity loss of the enzyme should be controlled for a viable application of the biosensor. Therefore, several of the known enzyme stabilizers, namely, bovine serum albumin (BSA) [23], glucose [24], and trehalose [25], were tested for their effect on the enzyme activity when immobilized in the paper. In addition, low concentration of sodium azide was also used to maintain sterility as all these stabilizers can be used as a carbon source by microorganisms. Adsorption of some such as enzymes, alkaline phosphatase (ALP) and horse radish peroxidase (HRP) has previously been reported to increase thermal stability of enzymes by 2-3 orders of magnitude. For example, ALP immobilized paper retained 60% of its activity after 48 hours of incubation at 60°C [26].

**Figure 5:**
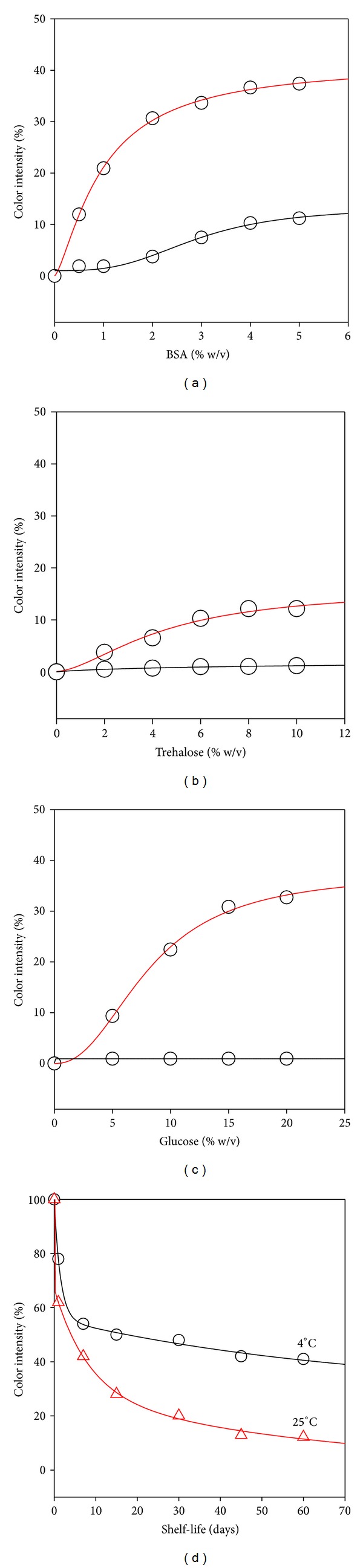
Effect of (a) BSA, (b) trehalose, (c) glucose, and (d) temperature on ChE activity. For (a), (b), and (c), black line is the color intensity for reaction mixture without enzyme, and red line is color intensity for reaction mixture with the enzyme.

The stability of the biosensor was attempted to be improved by adding BSA into sensor mixture at concentrations up to 5% (w/v). There was about a 40% increase in colour development for 5% BSA addition (Figure 5(a)). However, the negative controls with no enzyme exhibited slight yellow colour formation (approximately 10%). This nonspecific background reaction may be due to the sulfide moieties in BSA, and DTNB might be reacting and forming colour. The background colour development in control sensors can be confusing especially in visual determination. Thus it was concluded that BSA could not be used as an enzyme stabilizer in this biosensor development study. Next, effect of glucose on the stability of the biosensor was tested up to 15% (w/v) concentrations. It was found that glucose increased biosensor colour development by about 35% for 15% glucose addition (Figure 5(c)). There was no colour development in control sensors with the addition of glucose.

Trehalose is a dimer of two glucose molecules and has recently been shown to help retain the functional stability of enzymes [25]. Among sugars, trehalose has been known for its superior ability in protecting enzyme activity. Thus we tested the effect of trehalose on the stability of our biosensor up to 8% (w/v) (Figure 5(b)). The results showed that trehalose stabilized the enzyme and improved the stability by 10% for 10% of trehalose, but not as much as glucose. Thus glucose was chosen as the stabilizer compound for the rest of the studies. The final format of the biosensor included one additional compound for protection against microorganisms during storage. Sodium azide is an antibacterial agent, commonly used in the biosensor developments. In this study, 0.02% (w/v) sodium azide was used for its antibacterial effect, and the possible effect on the activity of the enzyme was experimented. 0.02% sodium azide did not have a negative effect on the performance of biosensor (results are not shown). Therefore it was concluded that sodium azide could be used as antibacterial agent in the biosensor.

The shelf-life of biosensors were tested after they were incubated at 4 or 25°C in nontransparent plastic bottles in order to prevent direct light exposure because both DTNB and ATCh are light sensitive molecules. As seen in Figure 5(d), the biosensor response to ATChI decreased to about 40% in one week at RT and to about 50% at 4°C when glucose was included in sensor mixture. The 4°C storage maintained the biosensor efficiency at a constant around 50% up to 60 days. Similarly, room temperature storage maintained around 20% activity.

**Figure 6:**
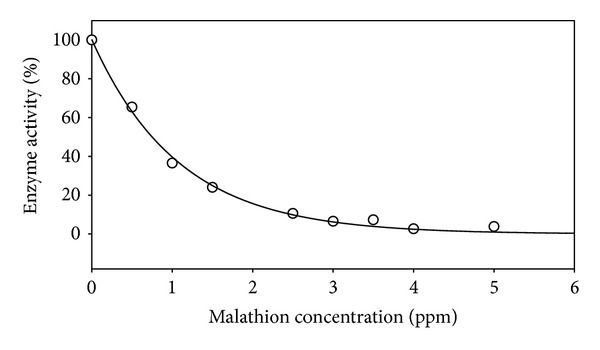
Relationship between inhibition of AChE activity and malathion.

For evaluating the ability of developed sensor for quantifying AChE inhibitory effects, a commonly used inhibitor, malathion, was used by preparing a standard paper sensor with 4 *μ*g/mL DNTB, 12 U/mL AChE, and 15% glucose (W/V). The mixture was adsorbed in the paper, dried, and used immediately. The biosensor response was tested by application of various concentrations of malathion samples with 4 *μ*g/mL ATChI final concentration. Malathion presence displayed inhibition of AChE proportional to its concentration (Figure 6). The inhibition curve approaches a constant value of 3% enzyme activity with malathion concentrations over 2.5 ppm. The limit of detection of the biosensor was determined by testing the inhibitory effect of malathion at a range of concentrations between 1 and 200 ppm. Above 2.5 ppm malathion, the biosensor response to ATChI was completely inhibited (Figure 6). Thus, limit of detection was determined to be 2.5 ppm for malathion. The enzyme activity as quantified with the biosensor did not reach zero value. This should be expected due to background colour development.

## 4. Conclusions

Performance of a dipstick-type acetylcholine esterase inhibitor sensor was investigated for determining optimal concentrations of sensor components and relevant enzyme stabilizers. The results in this study suggest that sugar stabilizers can be a potentially useful component in paper-based acetylcholine esterase inhibitor sensors. Among BSA, glucose, or trehalose, glucose was the best stabilizer in improving colour development and shelf-life at 4°C for especially visual tests. BSA improved the stability at higher levels compared to glucose, but the background levels did also increase to levels hindering visual quantification.
